# Dietary triacylglycerol hydroperoxide is not absorbed, yet it induces the formation of other triacylglycerol hydroperoxides in the gastrointestinal tract

**DOI:** 10.1016/j.redox.2022.102471

**Published:** 2022-09-14

**Authors:** Takumi Takahashi, Shunji Kato, Junya Ito, Naoki Shimizu, Isabella Supardi Parida, Mayuko Itaya-Takahashi, Masayoshi Sakaino, Jun Imagi, Kazuaki Yoshinaga, Aya Yoshinaga-Kiriake, Naohiro Gotoh, Ikuo Ikeda, Kiyotaka Nakagawa

**Affiliations:** a Laboratory of Food Function Analysis, Graduate School of Agricultural Science, Tohoku University, Miyagi, Japan; b J-Oil Mills Innovation Laboratory, Graduate School of Agricultural Science, Tohoku University, Miyagi, Japan; c Faculty of Food and Agricultural Sciences, Fukushima University, Fukushima, Japan; d Department of Life Science, Graduate School of Engineering Science, Akita University, Akita, Japan; e Department of Food Science and Technology, Tokyo University of Marine Science and Technology, Tokyo, Japan

**Keywords:** Triacylglycerol hydroperoxide, Intestinal absorption, Liquid chromatography-tandem mass spectrometry, Stable isotope-labeling, Oxidative stress

## Abstract

The *in vivo* presence of triacylglycerol hydroperoxide (TGOOH), a primary oxidation product of triacylglycerol (TG), has been speculated to be involved in various diseases. Thus, considerable attention has been paid to whether dietary TGOOH is absorbed from the intestine. In this study, we performed the lymph duct-cannulation study in rats and analyzed the level of TGOOH in lymph following administration of a TG emulsion containing TGOOH. As we successfully detected TGOOH from the lymph, we hypothesized that this might be originated from the intestinal absorption of dietary TGOOH [hypothesis I] and/or the *in situ* formation of TGOOH [hypothesis II]. To determine the validity of these hypotheses, we then performed another cannulation study using a TG emulsion containing a deuterium-labeled TGOOH (D2-TGOOH) that is traceable *in vivo*. After administration of this emulsion to rats, we clearly detected unlabeled TGOOH instead of D2-TGOOH from the lymph, indicating that TGOOH is not absorbed from the intestine but is more likely to be produced *in situ*. By discriminating the isomeric structures of TGOOH present in lymph, we predicted the mechanism by which the intake of dietary TGOOH triggers oxidative stress (*e.g.,* via generation of singlet oxygen) and induces *in situ* formation of TGOOH. The results of this study hereby provide a foothold to better understand the physiological significance of TGOOH on human health.

## 1 Introduction

**Fig. 1:**
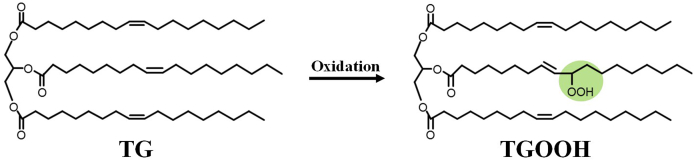
Chemical structures of triacylglycerol (TG) and TG hydroperoxide (TGOOH).

Lipids are essential nutrients that are widely contained in foods [1]. The major lipid in foods is triacylglycerol (TG), which consists of three fatty acids esterified to a glycerol backbone [2,3]. During excessive food processing and/or inadequate storage conditions, the fatty acid moieties of TG undergo radical (*e.g.,* thermal) and/or singlet oxygen (^1^O_2_) (*e.g.,* photo) oxidation to form TG hydroperoxide (TGOOH) (Fig. 1) [4]. Previously, our group and others have shown the presence of a small amount of TGOOH even in fresh edible oils [5] (*e.g.,* canola oil with a peroxide value (POV) of 0.8–2.0 meq/kg containing 10–50 nmol/mL of TGOOH [4]), which suggest that we may ingest a certain amount of TGOOH through daily food intake. Meanwhile, TGOOH is reportedly present *in vivo* (*e.g.,* in lipoproteins), and such TGOOH is speculated to be involved in the onset and progression of various diseases (*e.g.,* cardiovascular diseases) [6,7]. Due to these, considerable attention has been paid to the absorption of dietary TGOOH *in vivo*.

To the best of our knowledge, only a few studies have evaluated the absorption of TGOOH. For instance, Kanazawa et al. and Mohr et al. administered oils containing TGOOH (20 μmol [8] and 0.6 μmol [9], respectively) to rats; following this, however, the levels of TGOOH were below the detection limit in biological samples (*e.g.,* intestinal tract and lymph fluid). In line with these studies, Suomela et al. also reported that the levels of TGOOH in the intestinal tract of pigs fed highly oxidized oils (POV: 190 meq/kg) for two weeks were below detection limits [10]. These results collectively indicated that TGOOH is barely absorbed from the gastrointestinal tract. However, given the somewhat low sensitivity of the methods used in these studies [[8], [9], [10]] and the above indication of the presence of TGOOH *in vivo* [6,7], it is worthwhile to provide conclusive data regarding the absorption of dietary TGOOH using more sensitive methods to better understand its physiological significance *in vivo*.

**Fig. 2:**
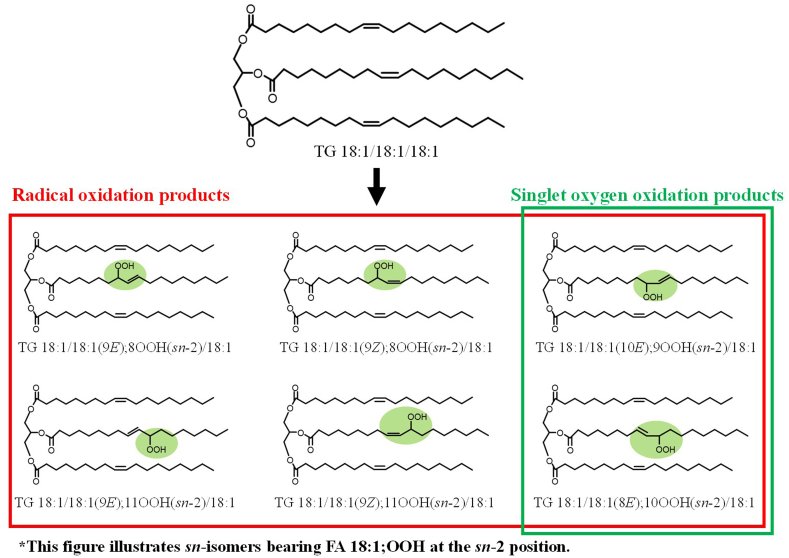
Chemical structures of TG 18:1/18:1/18:1 and its oxidation products. The hydroperoxyl group (OOH) position and the geometrical (*E/Z*) structure of TG 18:1/18:1/18:1; OOH depend on the oxidative stress involved (*i.e.,* radicals and/or singlet oxygen). The shorthand notation of lipids was in accordance with the LIPID MAPS nomenclature [55]. For instance, TG 18:1/18:1(9*E*); 8OOH(*sn*-2)/18:1 refers to 1, 3-dioleoyl-2-(8-hydroperoxy-9*E*-octadecamonoenoyl)-TG.

The conventional methods to detect TGOOH (*e.g.,* UV [8], chemiluminescence [9], and mass spectrometry methods [10]) have detection sensitivities ranging from μmol–pmol levels. Meanwhile, we recently developed a more sensitive method to analyze TGOOH using liquid chromatography-tandem mass spectrometry (HPLC-MS/MS), enabling detection at the fmol level [4]. Using this method, interestingly, we were able to detect TGOOH from the lymph fluid (in the order of fmol/μL) collected from rats given a TG emulsion containing 30 nmol of TGOOH (unpublished preliminary data). From this result, we deduced that a part of dietary TGOOH can be absorbed [hypothesis I]. Alternatively, it may also be possible that the ingested TGOOH triggered oxidative stress (*e.g.,* generation of reactive oxygen species such as free radicals and ^1^O_2_ [12]), leading to the *in situ* formation of TGOOH that we detected in the lymph [hypothesis II]. On top of that, our HPLC-MS/MS-based method is not only highly sensitive but also allows us to assess the oxidative stress (*i.e.,* radical or ^1^O_2_ oxidation) involved in TGOOH formation by discriminating the isomeric structure of TGOOH (Fig. 2) [4,11], such feature is considered to be useful in examining the validity of hypothesis I and/or II.

In this study, we first collected and analyzed lymph from rats following administration of a TG emulsion containing 32 nmol of TGOOH to confirm the reproducibility of our preliminary experiment. As we successfully detected TGOOH from the lymph, we then administered a TG emulsion containing a deuterium-labeled TGOOH (D2-TGOOH) that is traceable *in vivo*. By doing so, we aimed to determine the validity of the above hypotheses (*i.e.,* the presence of D2-TGOOH in the lymph would indicate the absorption of ingested TGOOH from the intestine [hypothesis I], while the presence of unlabeled TGOOH would suggest the *in situ* formation of TGOOH [hypothesis II]). The results obtained from this study will thereby elucidate the absorption of dietary TGOOH *in vivo*, which provides a foothold to understand the effects of TGOOH on human health.

## 2 Materials and methods

### 2.1 Reagents

Oleic acid (FA 18:1), trioleoyl glycerol (TG 18:1/18:1/18:1), pyridinium *p*-toluenesulfonate (PPTS), trioctanoyl glycerol (TG 8:0/8:0/8:0) and deuterated methanol (CH_3_OD) (99.5 atom % D) were obtained from Sigma-Aldrich (St. Louis, MO, USA). Dioleoyl glycerol (DG 18:1/18:1/0:0), 2-methoxypropene (MxP), *N,N′*-dicyclohexylcarbodiimide (DCC), dimethylaminopyridine (DMAP), 2, 2′-azobis (4-methoxy-2, 4-dimethylvaleronitrile) (MeO-AMVN), fatty acid-free bovine serum albumin and sodium methoxide were purchased from FUJIFILM Wako Pure Chemical Corporation (Osaka, Japan). Boron trifluoride diethyl ether complex was purchased from Tokyo Chemical Industry Co., Ltd. (Tokyo, Japan). Oleic acid methyl ester (FA 18:1; 1OMe) was purchased from Nu-Chek Prep Inc. (Elysian, MN). α-Lipase (neurase F3G) was kindly provided by Amano Enzyme Inc. (Aichi, Japan). Sodium taurocholate was purchased from Nacalai Tesque, Inc. (Kyoto, Japan). All other reagents were of analytical grade or higher.

### 2.2 Preparation of TG 18:1/18:1/18:1; OOH isomer references

MeO-AMVN (5 mg) was dissolved in chloroform (29 μL) and added to FA 18:1 (1 g). This mixture was incubated at 45 °C for 15 h. The generated six FA 18:1; OOH isomers consisting of OOH group positional and geometric (*E/Z*) isomers (*i.e.,* FA 18:1(9*Z*); 8OOH, FA 18:1(9*E*); 8OOH, FA 18:1(10*E*); 9OOH, FA 18:1(8*E*); 10OOH, FA 18:1(9*Z*); 11OOH and FA 18:1(9*E*); 11OOH) [13] were purified by semipreparative HPLC under the conditions used previously [4]. To refine the purity of the references, the obtained FA 18:1; OOH isomers were subjected to semipreparative HPLC again under the same conditions. The OOH group of each FA 18:1; OOH isomer was protected with MxP as previously described [4,14]. Each FA 18:1; OO-MxP isomer (5 mg) was dissolved in chloroform (462 μL) and esterified to DG 18:1/18:1/0:0 (9 mg) using DCC (3 mg) and DMAP (2 mg) [15]. The synthesized TG 18:1/18:1/18:1; OO-MxP isomers were purified by semipreparative HPLC using COSMOSIL 5C18-MS-II (5 μm, 10 × 250 mm, Nacalai Tesque, Inc.) with methanol–2-propanol (3:2, v/v) as the mobile phase (flow rate of 5 mL/min). The obtained isomers were deprotected as described previously [4,14]. The resultant TG 18:1/18:1/18:1; OOH isomers were lastly separated by normal-phase semipreparative HPLC using Inertsil SIL-100A (5 μm, 10 × 250 mm, GL Sciences Inc., Tokyo, Japan) with hexane–2-propanol (100:0.3, v/v) as the mobile phase (flow rate of 5 mL/min) to obtain their *sn*-isomers (*i.e.,* TG 18:1/18:1/18:1; OOH bearing FA 18:1; OOH isomers at *sn-*1 or *sn-*2 positions). The concentration of each reference was determined by measuring the concentration of FA 18:1 using the previously described gas chromatography method [4].

### 2.3 Preparation of TG 18:1/18:1/18:1[D2]; OOH isomer references

Preparation of FA 18:1[D2] was conducted according to previous studies [16,17]. FA 18:1; 1OMe (1 g) was refluxed at 105 °C for 30 min in CH_3_OD (10 mL) solution containing 3% (w/v) sodium methoxide. After cooling to room temperature, the solution was mixed with CH_3_OD (10 mL) containing 15% (v/v) boron trifluoride diethyl ether complex, and refluxed again for 10 min. After adding ethyl acetate and saturated sodium chloride solution, the upper layer (ethyl acetate layer) was collected. This layer was dehydrated with anhydrous sodium sulfate, filtered, and dried under nitrogen. To increase the incorporation of deuterium into FA 18:1; 1OMe, the dried extract was subjected to the same reaction four times. Based on gas chromatography-mass spectrometry analysis [16,17], the D2-labeling ratio of deuterated FA 18:1; 1OMe was higher than 95% (the remaining 5% was FA 18:1[D1]; 1OMe).

The obtained FA 18:1[D2]; 1OMe (1 g) was refluxed at 98 °C for 1 h in 142 mM sodium hydroxide solution (water–ethanol, 1:2, v/v) (119 mL). After cooling to room temperature, the solution was mixed with 10% (v/v) sulfuric acid (15 mL). After adding hexane and saturated sodium chloride solution, the upper layer (hexane layer) was collected. This layer was dehydrated with anhydrous sodium sulfate, filtered, and dried under nitrogen. A portion of this extract was dissolved in methanol and subjected to time-of-flight mass spectrometry (TOF/MS) (micrOTOF-Q II, Bruker Daltonics GmbH, Bremen, Germany) to confirm the generation of FA 18:1[D2] (*m/z* 307.2 [M+Na]^+^).

Using the synthesized FA 18:1[D2], each TG 18:1/18:1/18:1[D2]; OOH isomer was prepared by the same method as described above.

### 2.4 Analysis of TG 18:1/18:1/18:1; OOH and TG 18:1/18:1/18:1[D2]; OOH by HPLC-MS/MS

The synthesized references (*i.e.,* TG 18:1/18:1/18:1; OOH and TG 18:1/18:1/18:1[D2]; OOH) (1 μM) were dissolved in methanol containing 0.1 mM sodium acetate and directly infused (flow rate of 10 μL/min) into a 4000 QTRAP mass spectrometer (SCIEX, Tokyo, Japan). Precursor ions were checked by Q1 mass scan. Product ion detection conditions were set using the Analyst software (SCIEX, Tokyo, Japan). The optimized MS/MS parameters are shown in **SI 1**.

HPLC-MS/MS analysis was conducted with an ExionLC HPLC/UHPLC system (SCIEX, Tokyo, Japan) equipped with a 4000 QTRAP mass spectrometer according to a previously described method with slight modifications [4]. Samples were subjected to chromatographic separation using an analytical column (Inertsil SIL-100A; 5 μm, 2.1 × 250 mm; GL Sciences) attached to a cartridge guard column E (Inertsil SIL-100A; 5 μm, 1.5 × 10 mm; GL Sciences). Hexane–2-propanol–acetic acid (100:0.6:0.5, v/v/v) was used as the mobile phase (flow rate of 0.2 mL/min). To promote ionization, methanol–2-propanol (1:1, v/v) containing 0.2 mM sodium acetate (flow rate of 0.2 mL/min) was mixed with the eluate at the post column using a gradient mixer. The column temperature was set at 40 °C. Ionization conditions (electrospray-ionization: ESI) were set as follows: curtain gas, 20 psi; collision gas, 3 psi; ion spray voltage, 5500 V; temperature, 400 °C; ion source gas 1, 50 psi; ion source gas 2, 70 psi. The concentration of each TGOOH isomer in samples was determined based on external calibration curves obtained from the synthesized standards. Limit of quantification (LOQ) and the minimum concentration of which the coefficient value within ±20% (n=3) were determined according to a validation guideline [18].

### 2.5 Animals

All procedures were performed with protocols approved by the Tohoku University Ethics Review Board (approval number: 2019AgA-024). Male Sprague-Dawley rats (n=7, for experiment I; n=7, for experiment II; aged 8 weeks) weighing 290–320 g were obtained from CLEA Japan, Inc. (Tokyo, Japan). Prior to the experiments, all animals were acclimatized for 1 week. During this period, animals were caged and placed in the animal experimental room with controlled temperature and 12 h light/dark cycle, while given free access to water and commercial rodent chow (CE-2; CLEA Japan, Inc.).

### 2.6 Preparation of test lipid emulsions

The test lipid emulsion for experiment I (evaluation of lymphatic absorption of TG 18:1/18:1/18:1; OOH) was prepared from TG 18:1/18:1/18:1, sodium taurocholate and fatty acid-free bovine serum albumin. The test lipid emulsion for experiment II (evaluation of lymphatic absorption of TG 18:1/18:1/18:1[D2]; OOH) was prepared from TG 18:1/18:1/18:1, sodium taurocholate, fatty acid-free bovine serum albumin and synthesized TG 18:1/18:1/18:1[D2]; OOH references. Both samples were sonicated using a sonicator (55 W for 5 min, Sonifier Model 250, Branson Ultrasonics Corporation, MO, USA) [[19], [20], [21]].

### 2.7 Experiment I: evaluation of lymphatic absorption of TG 18:1/18:1/18:1; OOH

Lymph duct-cannulation surgery was performed according to a previously described method [22]. Briefly, a polyvinyl tube (single lumen clear vinyl tube SV-35; 0.5 mm I.D., 0.9 mm O.D.; Critchley Electrical Products, Auburn, Australia) and a polyethylene tube (single lumen polyethylene tube SP-55; 0.8 mm I.D., 1.2 mm O.D.; Critchley Electrical Products) were inserted into the thoracic lymphatic channel and stomach of the rats (n=7), respectively. After checking that the lymph fluid flowed stably (about 16 h after the surgery), the lymph was collected from the thoracic duct via the polyvinyl tube for 2 h (blank lymph). After the collection of the blank lymph, each rat was administered 3 mL of the test lipid emulsion consisting of TG 18:1/18:1/18:1 (200 mg), sodium taurocholate (200 mg), fatty acid-free bovine serum albumin (50 mg) and TG 18:1/18:1/18:1; OOH (32 nmol) into the stomach. A portion of the emulsion was collected before use. After the administration, lymph was collected at the following intervals; 0–0.5, 0.5–1, 1–2, 2–3, 3–4, 4–5, 5–6, 6–7, 7–8, 8–9 h. The collected lymph was stored at −80 °C until extraction.

The extraction of TG 18:1/18:1/18:1; OOH from the collected samples (*i.e.,* lymph and emulsion) was conducted using the modified Folch method [23,24]. Each sample (200 μL) was diluted with 0.9% (w/v) potassium chloride–1 mM ethylenediaminetetraacetic acid (200 μL). To this solution, chloroform–methanol (2:1, v/v) containing 0.002% (w/v) butylated hydroxytoluene was added (1.6 mL). The solution was then mixed using a vortex mixer and centrifuged (2000*g* for 20 min at 4 °C) to separate the upper layer (methanol–water layer) and the lipid-containing lower layer (chloroform layer). The lower layer was collected in cold test tubes. To the remaining upper layer, chloroform–methanol solution (10:1, v/v) was added (2 mL). This solution was mixed using a vortex mixer and centrifuged again (2000*g* for 20 min at 4 °C). The resultant lower layer (chloroform layer) was collected and combined with the previously collected lower layer. To this solution, TG 8:0/8:0/8:0 was added (2 mg). The solvent was then dried under nitrogen and reconstituted in hexane–2-propanol (1:1, v/v) (1 mL).

This lipid extract was purified by a Strata® SI-1 Silica cartridge (100 mg, 1 mL, Phenomenex Inc., CA, USA) as follows. The cartridge was washed with methanol (4 mL) and then equilibrated with hexane–2-propanol (1:1, v/v) (3 mL). The lipid extract (900 μL) and hexane–2-propanol (1:1, v/v) (3 mL) were loaded onto the cartridge. The resultant flow-through fraction was collected, evaporated and dissolved in hexane (1 mL). A portion of this solution (20 μL) was used for the analysis of TG 18:1/18:1/18:1; OOH.

The extraction recovery rates of TG 18:1/18:1/18:1; OOH from the lymph and the test lipid emulsion were checked as follows. The samples (blank lymph or emulsion) were spiked with 100 pmol of the synthesized TG 18:1/18:1/18:1; OOH standard. The extraction of TG 18:1/18:1/18:1; OOH was performed as described above. Extracts were analyzed by HPLC-MS/MS. Extraction recovery rates were calculated by comparison with the control (*i.e.,* blank lymph or emulsion that were not added with the standard) [25]. The results of the recovery test are shown in **SI 2**.

### 2.8 Experiment II: evaluation of lymphatic absorption of TG 18:1/18:1/18:1[D2]; OOH

Lymph duct-cannulation surgery was performed as in Experiment I. After the collection of the blank lymph, each rat (n=7) was administered 3 mL of the test lipid emulsion consisting of TG 18:1/18:1/18:1 (200 mg), sodium taurocholate (200 mg), fatty acid-free bovine serum albumin (50 mg), TG 18:1/18:1/18:1; OOH (87 nmol) and TG 18:1/18:1/18:1[D2]; OOH (24 nmol) into the stomach.

Extraction and analysis were performed as described above. The extraction recovery rates of TG 18:1/18:1/18:1[D2]; OOH from the lymph and the test lipid emulsion were checked as described above. The results are shown in **SI 3**.

## 3 Results and discussion

As previously mentioned, we recently developed a novel and highly sensitive HPLC-MS/MS-based method that enables the detection of TGOOH at the fmol level [4]. On top of that, this method also allows for the selective detection of different molecular species of TGOOH, including isomers with minor structural differences near the OOH group (*e.g.,* OOH positional isomers) that leads to assess the oxidative stress (*i.e.,* radical or ^1^O_2_ oxidation) [4,11]. Hence, we considered that this method may be a useful tool for obtaining more conclusive data regarding the absorption of dietary TGOOH.

**Fig. 3:**
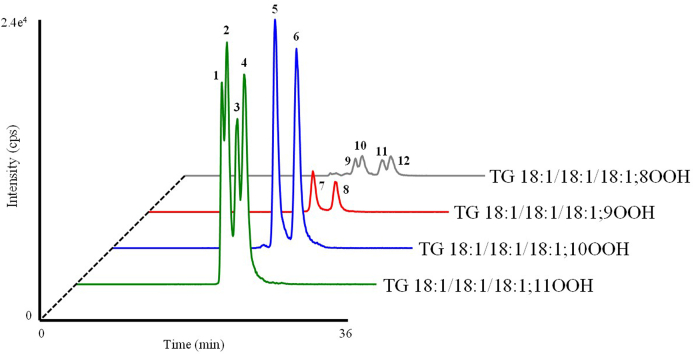
MRM chromatograms analyzing TG 18:1/18:1/18:1; OOH isomer standards (100 fmol each). (1) TG 18:1/18:1(9*Z*); 11OOH(*sn*-2)/18:1 (17.9 min); (2) TG 18:1/18:1(9*E*); 11OOH(*sn*-2)/18:1 (18.5 min); (3) TG 18:1/18:1(*sn*-2)/18:1(9*Z*); 11OOH (19.8 min); (4) TG 18:1/18:1(*sn*-2)/18:1(9*E*); 11OOH (20.6 min); (5) TG 18:1/18:1(8*E*); 10OOH(*sn*-2)/18:1 (20.0 min); (6) TG 18:1/18:1(*sn*-2)/18:1(8*E*); 10OOH (22.6 min); (7) TG 18:1/18:1(10*E*); 9OOH(*sn*-2)/18:1 (20.2 min); (8) TG 18:1/18:1(*sn*-2)/18:1(10*E*); 9OOH (22.9 min); (9) TG 18:1/18:1(9*Z*); 8OOH(*sn*-2)/18:1 (20.9 min); (10) TG 18:1/18:1(9*E*); 8OOH(*sn*-2)/18:1 (21.8 min); (11) TG 18:1/18:1(*sn*-2)/18:1(9*Z*); 8OOH (24.2 min); (12) TG 18:1/18:1(*sn*-2)/18:1(9*E*); 8OOH (25.3 min). The *sn*-position of FA 18:1; OOH within TG 18:1/18:1/18:1; OOH was determined by hydrolysis with α-lipase which act specifically at the *sn*-1 and *sn*-3 positions of TG [4]. The *E/Z* isomers were determined by monitoring the radical (*e.g.,* thermal) oxidation profile of TG 18:1/18:1/18:1 because TG 18:1/18:1/18:1(*E*); OOH isomers are characteristically formed under high temperature [4]. The retention time of each TG 18:1/18:1/18:1; OOH isomer was consistent with that of TG 18:1/18:1/18:1[D2]; OOH isomer (*c.f.,*Fig. 9).

In studying the absorption of TGOOH, we focused on TG 18:1/18:1/18:1; OOH which is one of the major TGOOH species in foods. TG 18:1/18:1/18:1, which is abundant in vegetable oils (*e.g.,* canola oil and olive oil [26]), consists of three FA 18:1 esterified to a glycerol backbone. TG 18:1/18:1/18:1 can be oxidized by radical and/or ^1^O_2_ to form twelve TG 18:1/18:1/18:1; OOH isomers that consist of the OOH positional, *E/Z* and *sn*-isomers (Fig. 2) [13]. To accurately quantify the level of these isomers, we prepared standard compounds corresponding to each TG 18:1/18:1/18:1; OOH isomer. By analyzing the standards using the aforementioned HPLC-MS/MS method, we found that each isomer produces a characteristic product ion at the OOH group by the addition of sodium (*e.g.,* loss of 116 Da for TG 18:1/18:1/18:1; 11OOH) [[27], [28], [29], [30]]. Based on these product ions, the multiple reaction monitoring (MRM) pairs were determined (*e.g., m/z* 939.8 > 823.8 for TG 18:1/18:1/18:1; 11OOH isomers) (**SI 1**). Using a silica-based column that allows for the effective separation of *E/Z* and *sn*-isomers under normal-phase HPLC [4], we were able to separate and selectively detect the twelve TG 18:1/18:1/18:1; OOH isomers (Fig. 3). Overall, to the best of our knowledge, this method is able to detect TGOOH with the LOQ of 1–10 fmol/injection, which is the most sensitive among the reported methods for TGOOH [4,[8], [9], [10]]. Therefore, this method was considered capable of sensitively quantifying the twelve TG 18:1/18:1/18:1; OOH isomers in samples (*i.e.,* a test lipid emulsion and lymph) from the following experiments.

**Fig. 4:**
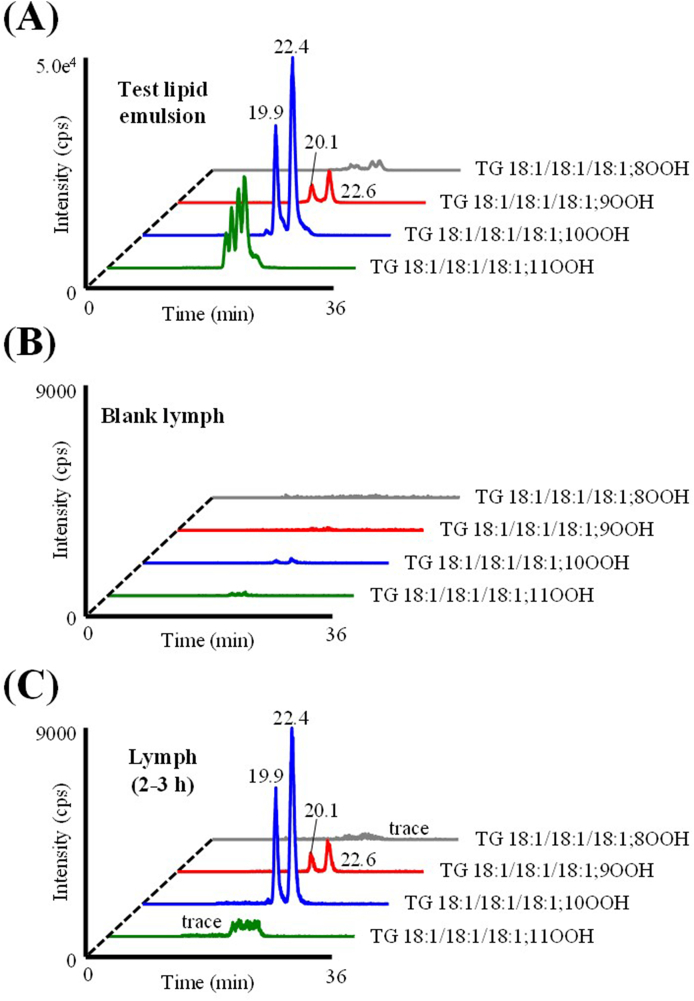
Representative MRM chromatograms analyzing the test lipid emulsion (A) and lymph samples (B and C).

As mentioned in the Introduction, we were able to detect TGOOH in the lymph collected from a small number of rats administered a TG emulsion containing 30 nmol of TGOOH (unpublished preliminary data). To confirm the reproducibility of the experiment, we performed the present study (Experiment I) under similar conditions but with a larger sample size (n=7). To prepare the TG emulsion, we mixed TG 18:1/18:1/18:1 with distilled water containing sodium taurocholate and fatty acid-free bovine serum albumin and sonicated the mixture [19,20]. The resultant emulsion, which was then used as the test sample in Experiment I (Fig. 4A), fortuitously contained 32 nmol of TG 18:1/18:1/18:1; OOH. This may be due to the fact that the commercial TG 18:1/18:1/18:1 standard used to prepare the TG emulsion originally contained a small amount of TG 18:1/18:1/18:1; OOH (data not shown). Also, the heat generated during the sonication might have caused oxidation of TG 18:1/18:1/18:1 [31], to yield an emulsion with the above characteristic (*i.e.,* containing 32 nmol of TG 18:1/18:1/18:1; OOH).

**Fig. 5:**
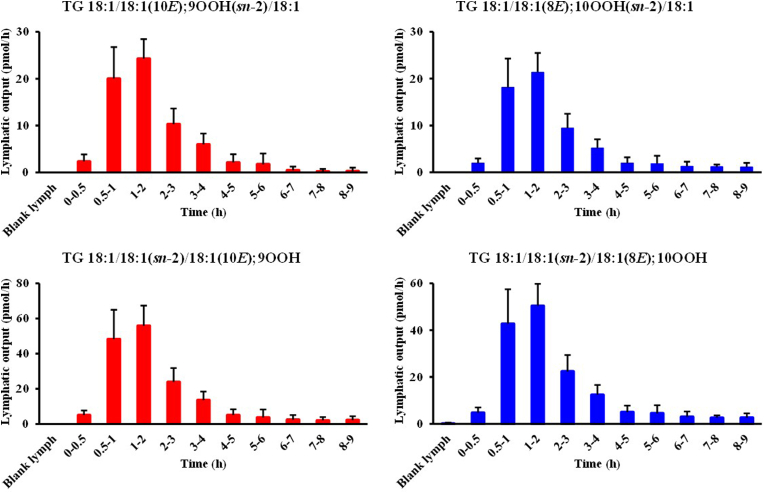
Time-dependent changes (0–0.5, 0.5–1, 1–2, 2–3, 3–4, 4–5, 5–6, 6–7, 7–8, and 8–9 h) of TG 18:1/18:1/18:1; 9OOH and TG 18:1/18:1/18:1; 10OOH species in the lymph after administration of the test lipid emulsion in lymph-cannulated rats. Values are indicated as means ± standard deviations (n=7). This figure shows the results of Experiment I.

**Fig. 6:**
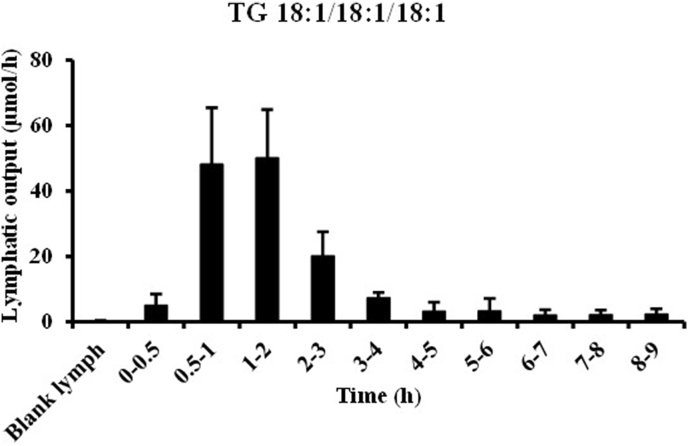
Time-dependent changes (0–0.5, 0.5–1, 1–2, 2–3, 3–4, 4–5, 5–6, 6–7, 7–8, and 8–9 h) of TG 18:1/18:1/18:1 in the lymph after administration of the test lipid emulsion in lymph-cannulated rats. Values are indicated as means ± standard deviations (n=7). TG 18:1/18:1/18:1 was analyzed using HPLC-MS consisting of a Nexera LC system (Shimadzu Corp., Kyoto, Japan) equipped with a TOF/MS. The extracted samples were 500–fold diluted and a portion (1 μL) was separated with the use of an ACQUITY UPLC HSS C18 column (1.8 μm, 1.0 × 150 mm; Waters, MA, USA) with acetonitrile–1-propanol (4:1, v/v) containing acetic acid (0.1%, v/v) as the mobile phase (flow rate of 0.175 mL/min). The column temperature was set at 40 °C. ESI was used as an ion source with the following experimental parameters: capillary, 4100 V; dry gas, 1.6 bar; dry temp, 180 °C; collision energy, 10 eV; collision RF, 920 Vpp; transfer time, 95 μs; pre pulse storage, 15.0 μs. TG 18:1/18:1/18:1 was detected in the extracted ion chromatogram (*m/z* 907.8 [M+Na]^+^) and its concentration was determined based on external calibration curves (0.1–10 pmol) obtained from a commercial standard.

After the lymph-cannulation surgery, lymph was collected from the thoracic duct for 2 h (blank lymph) before the administration of the test emulsion [22]. As shown in Fig. 4B, TG 18:1/18:1/18:1; OOH isomers were barely detected in the blank lymph, suggesting that lymph does not originally contain any TG 18:1/18:1/18:1; OOH isomers. Following the administration of the emulsion, we clearly detected TG 18:1/18:1/18:1; 9OOH and TG 18:1/18:1/18:1; 10OOH, among other TG 18:1/18:1/18:1; OOH isomers, in the lymph (Fig. 4C). To the best of our knowledge, this is the first study that detected such TGOOH in the lymph after gastric administration of TGOOH mostly owing to our highly sensitive and selective HPLC-MS/MS-based analytical method. In addition, our extraction method allows for the efficient isolation of TGOOH from lymph (extraction recovery >80% (**SI 2**)), enabling better detection of TGOOH. Fig. 5 shows the time-dependent changes in the concentration of TG 18:1/18:1/18:1; OOH (*i.e.,* TG 18:1/18:1/18:1; 9OOH and TG 18:1/18:1/18:1; 10OOH species) in the lymph. According to this data, the lymphatic concentrations of TGOOH isomers reached their highest at 1–2 h following the administration of TG emulsion. Also, since the emulsion contained a substantial amount of unoxidized TG 18:1/18:1/18:1 whose level in lymph reached its peak at 1–2 h following administration of test emulsion (Fig. 6) [19,20], we deduced that dietary TGOOH can be absorbed from the intestine in the same manner as TG [hypothesis I]. However, considering that only certain TGOOH isomers were present in the lymph and results of previous studies that administered other lipid hydroperoxides to animals [32,33], it is also possible that the ingested TG 18:1/18:1/18:1; OOH triggered oxidative stress (*e.g.,* generation of free radicals and/or ^1^O_2_) that led to the *in situ* formation of TG 18:1/18:1/18:1; 9OOH and TG 18:1/18:1/18:1; 10OOH [hypothesis II]. Therefore, we aimed to determine the validity of the above hypotheses using a deuterium-labeled TGOOH (D2-TGOOH) that is traceable *in vivo*. In this case, the presence of D2-TGOOH in the lymph would indicate the absorption of ingested TGOOH from the intestine [hypothesis I], while the presence of unlabeled TGOOH would suggest the *in situ* formation of TGOOH [hypothesis II]).

**Fig. 7:**
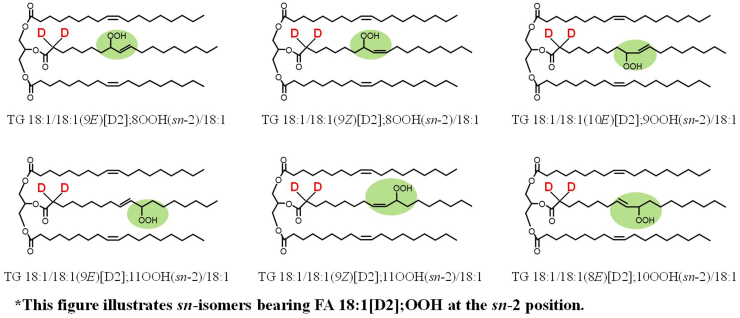
Chemical structures of TG 18:1/18:1/18:1[D2]; OOH isomers. The shorthand notation of lipids was in accordance with the LIPID MAPS nomenclature [55].

**Fig. 8:**
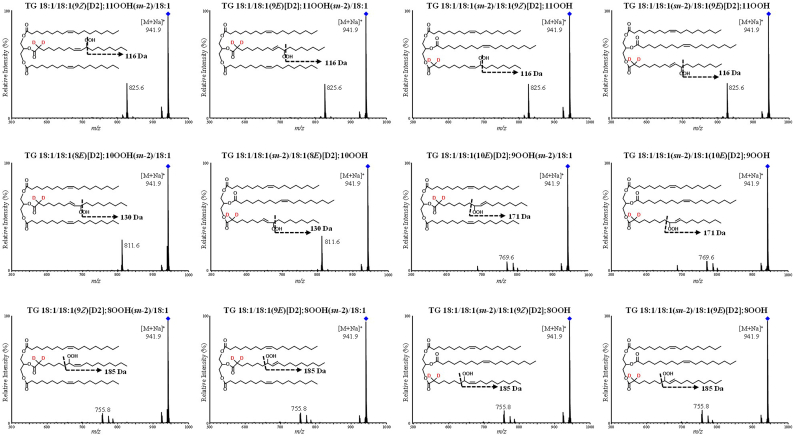
The product ion mass spectra of reference TG 18:1/18:1/18:1[D2]; OOH isomers.

To begin with, we synthesized FA 18:1[D2] with deuterium introduced at the α-carbon of the carbonyl group [16,17] and prepared twelve TG 18:1/18:1/18:1[D2]; OOH isomer standards (Fig. 7) in the same manner as unlabeled TG 18:1/18:1/18:1; OOH. When the standards were analyzed using HPLC-MS/MS, TG 18:1/18:1/18:1[D2]; OOH isomers generated specific product ions by the addition of sodium (*e.g.,* loss of 116 Da for TG 18:1/18:1/18:1[D2]; 11OOH (Fig. 8)) [[27], [28], [29], [30]]. Based on these results, we confirmed that these fragmentations occur near the OOH group by α-cleavage [30] just like the unlabeled TG 18:1/18:1/18:1; OOH and that the deuterium located at the α-carbon of the carbonyl group does not affect the fragmentation patterns. Using a normal-phase HPLC, we were able to separate and selectively detect the twelve TG 18:1/18:1/18:1[D2]; OOH isomers. On top of that, this method is able to detect D2-TGOOH with the LOQ of 1–10 fmol/injection. To the best of our knowledge, this study is the first to synthesize stable isotope-labeled TGOOH standards and develop an accurate method for their analysis.

**Fig. 9:**
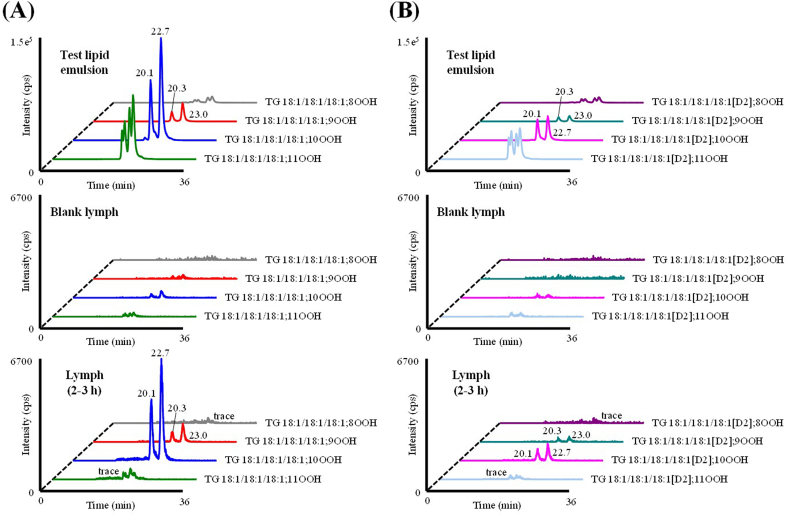
Representative MRM chromatograms of TG 18:1/18:1/18:1; OOH (A) and TG 18:1/18:1/18:1[D2]; OOH (B).

**Fig. 10:**
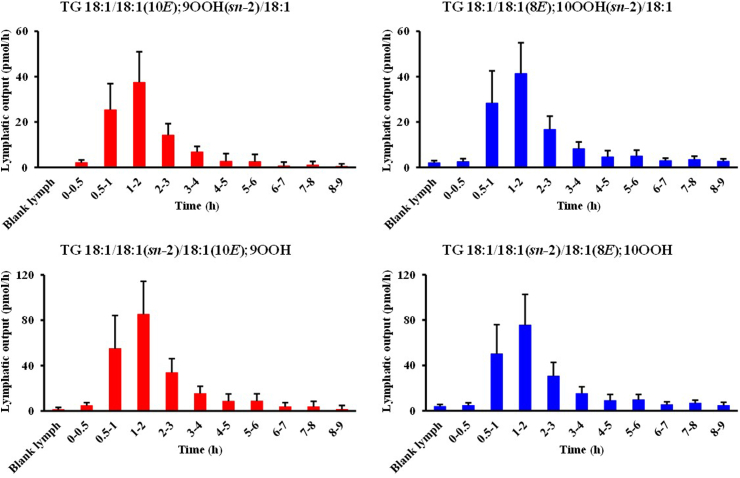
Time-dependent changes (0–0.5, 0.5–1, 1–2, 2–3, 3–4, 4–5, 5–6, 6–7, 7–8, and 8–9 h) of TG 18:1/18:1/18:1; 9OOH and TG 18:1/18:1/18:1; 10OOH isomers in the lymph after administration of the test lipid emulsion in lymph-cannulated rats. Values are indicated as means ± standard deviations (n=7). This figure shows the results of Experiment II.

Next, we prepared a TG emulsion for Experiment II by mixing TG 18:1/18:1/18:1 and the synthesized TG 18:1/18:1/18:1[D2]; OOH with distilled water containing sodium taurocholate and fatty acid-free bovine serum albumin before sonicating the mixture [19,20]. The resultant emulsion, which was then used as the test sample in Experiment II (Fig. 9), contained 87 nmol of TG 18:1/18:1/18:1; OOH and 24 nmol of TG 18:1/18:1/18:1[D2]; OOH. The level of unlabeled TG 18:1/18:1/18:1; OOH in this emulsion was higher than the one used in Experiment I, most likely due to the higher degree of TG 18:1/18:1/18:1 oxidation that occurred during the preparation steps (*e.g.,* during sonication). However, in line with this change, we found that the lymphatic concentration of TG 18:1/18:1/18:1; OOH was also increased compared to the result from Experiment I (*c.f.,* Fig. 10), thus, we believe that the HPLC-MS/MS measurements of TGOOH were accurate in both experiments. Following the administration of the test emulsion, we clearly detected TG 18:1/18:1/18:1; 9OOH and TG 18:1/18:1/18:1; 10OOH from the lymph (Fig. 9A). Also, the lymphatic concentrations of these isomers reached their highest at 1–2 h following the administration (Fig. 10), which were consistent with our findings in Experiment I. While we barely detected TG 18:1/18:1/18:1[D2]; OOH isomers in the blank lymph, we observed a slight increase in their levels (*e.g.,* TG 18:1/18:1/18:1[D2]; 9OOH and TG 18:1/18:1/18:1[D2]; 10OOH) following the administration of TG 18:1/18:1/18:1[D2]; OOH at a level that was approximately similar to that administered in Experiment I (Fig. 9B). However, since there is only a mass difference of 2 Da between D2 and unlabeled isotopic pairs, it is likely that most of these signals were derived from the unlabeled TG 18:1/18:1/18:1; OOH isotopes (**SI 4**) [34]. In fact, when the signals of unlabeled isotope isomers were subtracted [35], the concentrations of all TG 18:1/18:1/18:1[D2]; OOH isomers were under the LOQ (1–10 fmol/injection). Hence, the above results (*i.e.,* the presence of unlabeled TGOOH instead of D2-TGOOH in the lymph) indicated that TGOOH is not absorbed from the intestine but is likely to be produced *in situ*.

Since we confirmed that TGOOH is not absorbed in its intact form, this suggests the possibility that TGOOH undergoes degradation in the gastrointestinal tract to yield breakdown products that can be absorbed from the intestine. In fact, some previous studies have reported that lipid hydroperoxides are efficiently reduced to hydroxyl species in the gastrointestinal tract [36,37]. Therefore, to confirm whether such phenomenon occurs in the present study, we incubated TGOOH in rat gastric and small intestinal mucosa homogenates and analyzed the formation of the hydroxyl species (TGOH) using HPLC-MS/MS. After 30 min of incubation, we found that the TGOOH level decreased to around 20% (**SI 5A**) while the level of TGOH significantly increased (**SI 5B**). These results suggested that TGOOH decomposes rapidly (*e.g.,* through reductive degradation) in the gastrointestinal environment. More importantly, we clearly detected TGOH in the lymph sample collected in Experiment I (**SI 6C**). This TGOH may derive from the intestinal absorption of TGOH and/or the *in situ* formation of TGOH, and thus, we tried to trace the D2-TGOH in the lymph samples collected in Experiment II. Interestingly, we did not detect D2-TGOH in the lymph (**SI 6D**). Altogether, these data suggest that TGOH is not absorbed from the intestine but is most likely present as the reduction product of the *in situ* formed TGOOH. The possible mechanism of TGOOH formation is explained in the following paragraph.

**Fig. 11:**
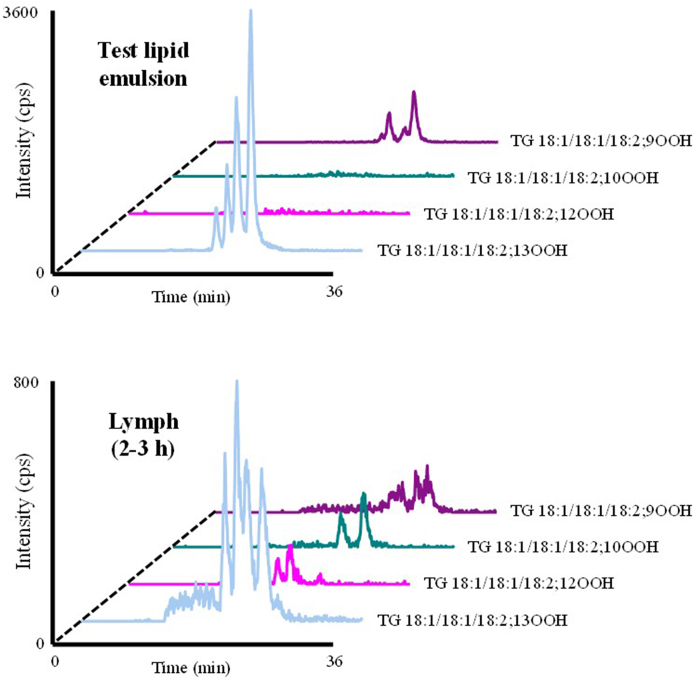
Representative MRM chromatograms analyzing the test lipid emulsion and lymph.

As already mentioned, since radical and ^1^O_2_ oxidation yield characteristically different lipid hydroperoxide isomers [13], we can assess the oxidative stress involved in TGOOH formation based on its isomeric structure [4,11]. In the present study, TG 18:1/18:1/18:1; 9OOH and TG 18:1/18:1/18:1; 10OOH were the predominant isomers detected in the lymph, indicating that ^1^O_2_ oxidation is likely to be involved in TGOOH formation. This reaction may be triggered by the intake of TGOOH. Previously, dietary lipid hydroperoxides have been reported to cause oxidative stress that induces pro-inflammatory changes in the gastrointestinal tract [32,33,38,39]. Separately, other studies reported that an inflamed intestinal mucosa is highly infiltrated with neutrophils (*e.g.,* in ulcerative colitis patients [40,41]), which generate ^1^O_2_ via the myeloperoxidase system of activated phagocytosis as the initial defense mechanism against tissue damage [[42], [43], [44]]. From these facts, we deduce that the administered TGOOH might trigger oxidative stress by generating ^1^O_2_ via neutrophil infiltration into the gastrointestinal tract, which subsequently induced ^1^O_2_ oxidation of TG *in situ*. To further validate this assertion, we analyzed other TGOOH molecular species such as TG 18:1/18:1/18:2; OOH [4] because the lymph contained a relatively large amount of unoxidized TG 18:1/18:1/18:2 (15%) (the remaining 85% was TG 18:1/18:1/18:1) (data not shown). As a result, in support of our findings, we found ^1^O_2_ oxidation-specific isomers (*i.e.,* TG 18:1/18:1/18:2; 10OOH and TG 18:1/18:1/18:2; 12OOH [4]) in the lymph, despite the emulsion containing undetectable levels of these isomers (Fig. 11). These findings may serve as an important basis to formulate effective strategies to prevent and treat various diseases. For instance, considering that continuous accumulation of lipid hydroperoxides *in vivo* has been linked to the onset and progression of diseases (*e.g.,* atherosclerosis [27], diabetes [45] and Alzheimer's disease [46]), the use of antioxidants (*e.g.,* as supplements and food additives) that specifically inhibit ^1^O_2_ oxidation, such as carotenoids [47] may be useful for attenuating *in situ* formation of TGOOH, thus reducing the risk of diseases. To substantiate this, further works are needed to elucidate the association of TGOOH in the development of diseases.

This study has two main limitations. First, we could only trace a small portion of the degradation products that may be formed from the administered TGOOH. Based on the reports by Kanazawa et al. [8,48] and other investigators that proposed the degradation pathway of lipid hydroperoxides [37,49,50], it appears that TGOOH is degraded in the gastrointestinal tract to yield breakdown products such as aldehydes. Since such compounds appear to be transported into the systemic circulation [48,51,52], future studies should broaden the scope of analysis to include other biological samples (*e.g.,* blood and liver). Second, we were unable to perform experiments using a wider range of TGOOH doses that is achievable through daily food intake. The actual dietary intake of TGOOH in humans may be higher than the doses we used in the present study, and thus, future studies should perform under such conditions to strengthen the cause-effect relationship. Such data will help to clarify the mechanisms of the dose-dependent relationship between the intake amount of lipid hydroperoxides and the degree of oxidative stress damage in the intestine which leads to various diseases [53,54]. Nonetheless, as far as the present study goes, we successfully achieved our main goal of elucidating the absorption of TGOOH owing to the use of the isotope-labeling technique and our highly sensitive and selective HPLC-MS/MS-based method.

## 4 Conclusions

In this study, we investigated the absorption of TGOOH by performing lymph-cannulation and utilizing our novel HPLC-MS/MS method for the quantification of lymphatic TGOOH levels. As a result, we found that dietary TGOOH is not likely to be absorbed from the intestine. Rather, the presence of TGOOH in lymph is more likely due to the *in situ* formation of TGOOH, presumably via ^1^O_2_ oxidation of TG triggered by the administered TGOOH. Altogether, these findings provide a foothold to better understand the effects of TGOOH on human health.

## Declaration of competing interest

The authors declare no conflicts of interest.
