# Supplementary material for: Dietary triacylglycerol hydroperoxide is not absorbed, yet it induces the formation of other triacylglycerol hydroperoxides in the gastrointestinal tract
Source: Redox Biol. 2022 Sep 14;57:102471. doi: 10.1016/j.redox.2022.102471 (PMC9493066; doi:10.1016/j.redox.2022.102471)
Supplement: Multimedia component 1 [file mmc1.docx]

**SI 1.** MS/MS parameters for TG 18:1/18:1/18:1;OOH and TG 18:1/18:1/18:1[D2];OOH isomers.

(1) TG 18:1/18:1/18:1;8OOH; (2) TG 18:1/18:1/18:1;9OOH; (3) TG 18:1/18:1/18:1;10OOH; (4) TG 18:1/18:1/18:1;11OOH;

(5) TG 18:1/18:1/18:1[D2];8OOH; (6) TG 18:1/18:1/18:1[D2];9OOH; (7) TG 18:1/18:1/18:1[D2];10OOH; (8) TG 18:1/18:1/18:1[D2];11OOH

|  | TG 18:1/18:1/18:1;OOH | | | | TG 18:1/18:1/18:1[D2];OOH | | | |
| --- | --- | --- | --- | --- | --- | --- | --- | --- |
|  | (1) | (2) | (3) | (4) | (5) | (6) | (7) | (8) |
| Q1 | 939.8 | | | | 941.9 | | | |
| Q3 | 754.0 | 767.7 | 809.7 | 823.8 | 755.8 | 769.6 | 811.6 | 825.6 |
| DP（V） | 60 | 48 | 55 | 48 | 26 | 26 | 29 | 29 |
| EP（V） | 10 | | | | 10 | | | |
| CE（V） | 43 | 43 | 49 | 52 | 42 | 42 | 54 | 50 |
| CXP（V） | 17 | 11 | 12 | 12 | 17 | 17 | 19 | 21 |

DP: Declustering potential, EP: Entrance potential, CE: Collision energy, CXP: Collision cell exit potential

**SI 2.** Extraction recovery rates of TG 18:1/18:1/18:1;OOH from lymph and test lipid emulsion. Values are indicated as means ± standard deviations (n=4).

(1) TG 18:1/18:1(9*Z*);11OOH(*sn*-2)/18:1; (2) TG 18:1/18:1(9*E*);11OOH(*sn*-2)/18:1; (3) TG 18:1/18:1(*sn*-2)/18:1(9*Z*);11OOH; (4) TG 18:1/18:1(*sn*-2)/18:1(9*E*);11OOH; (5) TG 18:1/18:1(8*E*);10OOH(*sn*-2)/18:1; (6) TG 18:1/18:1(*sn*-2)/18:1(8*E*);10OOH; (7) TG 18:1/18:1(10*E*);9OOH(*sn*-2)/18:1; (8) TG 18:1/18:1(*sn*-2)/18:1(10*E*);9OOH; (9) TG 18:1/18:1(9*Z*);8OOH(*sn*-2)/18:1; (10) TG 18:1/18:1(9*E*);8OOH(*sn*-2)/18:1; (11) TG 18:1/18:1(*sn*-2)/18:1(9*Z*);8OOH; (12) TG 18:1/18:1(*sn*-2)/18:1(9*E*);8OOH

| % |  | (1) | | |  | (2) | | |  | (3) | | |  | (4) | | |  | (5) | | |  | (6) | | |
| --- | --- | --- | --- | --- | --- | --- | --- | --- | --- | --- | --- | --- | --- | --- | --- | --- | --- | --- | --- | --- | --- | --- | --- | --- |
| Lymph |  | 98.9 | ± | 9.8 |  | 96.8 | ± | 1.5 |  | 88.2 | ± | 5.9 |  | 97.1 | ± | 5.8 |  | 91.8 | ± | 2.2 |  | 82.9 | ± | 8.7 |
| Emulsion |  | 90.8 | ± | 3.4 |  | 93.3 | ± | 5.4 |  | 92.6 | ± | 3.9 |  | 93.6 | ± | 4.8 |  | 93.9 | ± | 3.6 |  | 86.7 | ± | 5.6 |
|  |  |  |  |  |  |  |  |  |  |  |  |  |  |  |  |  |  |  |  |  |  |  |  |  |
| % |  | (7) | | |  | (8) | | |  | (9) | | |  | (10) | | |  | (11) | | |  | (12) | | |
| Lymph |  | 95.1 | ± | 3.5 |  | 83.5 | ± | 8.4 |  | 93.4 | ± | 7.8 |  | 90.5 | ± | 6.2 |  | 86.2 | ± | 6.5 |  | 83.2 | ± | 4.8 |
| Emulsion |  | 91.8 | ± | 5.0 |  | 85.7 | ± | 5.5 |  | 93.6 | ± | 6.2 |  | 94.0 | ± | 7.3 |  | 88.7 | ± | 7.4 |  | 83.7 | ± | 8.3 |

**SI 3.** Extraction recovery rates of TG 18:1/18:1/18:1[D2];OOH from lymph and test lipid emulsion. Values are indicated as means ± standard deviations (n=3–4).

(1) TG 18:1/18:1(9*Z*)[D2];11OOH(*sn*-2)/18:1; (2) TG 18:1/18:1(9*E*)[D2];11OOH(*sn*-2)/18:1; (3) TG 18:1/18:1(*sn*-2)/18:1(9*Z*)[D2];11OOH; (4) TG 18:1/18:1(*sn*-2)/18:1(9*E*)[D2];11OOH; (5) TG 18:1/18:1(8*E*)[D2];10OOH(*sn*-2)/18:1; (6) TG 18:1/18:1(*sn*-2)/18:1(8*E*)[D2];10OOH; (7) TG 18:1/18:1(10*E*)[D2];9OOH(*sn*-2)/18:1; (8) TG 18:1/18:1(*sn*-2)/18:1(10*E*)[D2];9OOH; (9) TG 18:1/18:1(9*Z*)[D2];8OOH(*sn*-2)/18:1; (10) TG 18:1/18:1(9*E*)[D2];8OOH(*sn*-2)/18:1; (11) TG 18:1/18:1(*sn*-2)/18:1(9*Z*)[D2];8OOH; (12) TG 18:1/18:1(*sn*-2)/18:1(9*E*)[D2];8OOH

| % |  | (1) | | |  | (2) | | |  | (3) | | |  | (4) | | |  | (5) | | |  | (6) | | |
| --- | --- | --- | --- | --- | --- | --- | --- | --- | --- | --- | --- | --- | --- | --- | --- | --- | --- | --- | --- | --- | --- | --- | --- | --- |
| Lymph |  | 91.3 | ± | 6.5 |  | 92.8 | ± | 2.9 |  | 90.8 | ± | 2.6 |  | 94.9 | ± | 1.2 |  | 88.2 | ± | 2.1 |  | 90.2 | ± | 2.3 |
| Emulsion |  | 91.5 | ± | 6.9 |  | 92.1 | ± | 7.3 |  | 86.1 | ± | 8.5 |  | 91.3 | ± | 6.4 |  | 93.1 | ± | 7.0 |  | 88.2 | ± | 6.2 |
|  |  |  |  |  |  |  |  |  |  |  |  |  |  |  |  |  |  |  |  |  |  |  |  |  |
| % |  | (7) | | |  | (8) | | |  | (9) | | |  | (10) | | |  | (11) | | |  | (12) | | |
| Lymph |  | 89.8 | ± | 2.3 |  | 89.1 | ± | 1.4 |  | 94.1 | ± | 3.5 |  | 84.6 | ± | 3.6 |  | 83.1 | ± | 4.8 |  | 85.1 | ± | 1.4 |
| Emulsion |  | 95.6 | ± | 6.9 |  | 88.3 | ± | 6.2 |  | 98.6 | ± | 9.0 |  | 88.6 | ± | 3.7 |  | 92.0 | ± | 8.6 |  | 86.2 | ± | 6.8 |

**SI 4.** MRM chromatograms analyzing TG 18:1/18:1/18:1;OOH isomer standards (250 fmol each).

The MRM of TG 18:1/18:1/18:1[D2];OOH partially includes the natural isotope peaks of unlabeled TG 18:1/18:1/18:1;OOH due to the presence of natural isotopes (*e.g.,* ^13^C (1.07%), ^17^O (0.04%) and ^2^H (0.01%) [J.R. De Laeter, J.K. Böhlke, P. de Bièvre, H. Hidaka, H.S. Peiser, K.J.R. Rosman, P.D.P. Taylor, Atomic weight of the elements: Review 2000, Pure Appl. Chem. 75 (2003) 683–800.]).

**SI 5.** The relative amount of TG 18:1/18:1/18:1;10OOH (A) and TG 18:1/18:1/18:1;OH (B) after incubation of test lipid emulsion containing TG 18:1/18:1/18:1;OOH with gastric and small intestinal mucosa homogenates. (○) Control (incubation without homogenate); (■) with the small intestinal homogenate; (▲) with the gastric homogenate. Values are indicated as means ± standard deviations (n=3). Only TG 18:1/18:1/18:1;10OOH is shown here as a representative for TG 18:1/18:1/18:1;OOH isomers. TG 18:1/18:1/18:1;OH was calculated as the total OH positional, *E/Z* and *sn*-isomers.

Rat gastric and small intestinal mucosa homogenates were prepared according to a previous report [36]. These homogenates (final concentration 5 mg protein/mL) or Tris-HCl buffer (0.1 M, pH 7.4, containing 0.135 M KCl) were each mixed with the test lipid emulsion containing TG 18:1/18:1/18:1;OOH. A portion of the emulsion was collected before incubation (0 min). The mixture was incubated at 37°C in an oscillating water bath (60 cycles per min; Taitec Personal-11; Taitec Corporation, Saitama, Japan). After the incubation (30, 60, and 90 min), the samples were collected. A portion of the sample extract (20 µL) was separated using the COSMOSIL 5C18-MS-II (5 µm, 2.0 × 250 mm, Nacalai Tesque, Inc.) with methanol–2-propanol (3:1, v/v) as the mobile phase (flow rate of 0.2 mL/min). The column temperature was set at 50°C. ESI was used as an ion source with the following experimental parameters: curtain gas, 20 psi; collision gas, 5 psi; ion spray voltage, 5000 V; temperature, 500°C; ion source gas 1, 40 psi; ion source gas 2, 40 psi. TG 18:1/18:1/18:1;10OOH and TG 18:1/18:1/18:1;OH were detected in the MRM mode (*m/z* 939.8 > 809.7 for TG 18:1/18:1/18:1;10OOH and *m/z* 923.9 > 625.6 for TG 18:1/18:1/18:1;OH). This experiment was performed with protocols approved by the Tohoku University Ethics Review Board (approval number: 2022AgA-014).

**SI 6.** Representative MRM chromatograms from analysis of TG 18:1/18:1/18:1;OH and TG 18:1/18:1/18:1[D2];OH standards (A and B), and lymph samples from Experiment I and II (C and D).

TG 18:1/18:1/18:1;OH and TG 18:1/18:1/18:1[D2];OH were analyzed by the same method as shown in **SI 5**. To confirm the retention time of TG 18:1/18:1/18:1;OH and TG 18:1/18:1/18:1[D2];OH, their standard compounds were prepared by reducing the standard TG 18:1/18:1/18:1;OOH and TG 18:1/18:1/18:1[D2];OOH with NaBH_4_ [36].
